# Pharmacokinetics of Mitomycin C Following Hepatic Arterial Chemoembolization With Gelfoam

**DOI:** 10.1155/1992/67843

**Published:** 1992

**Authors:** Jin Wen Ding, Zai de Wu, Roland Andersson, Stig Bengmark

**Affiliations:** 1 Department of Surgery Tongji Hospital Tongji Medical University Wuhan 430030 China; 2 Department of Surgery Lund University Lund S-221 85 Sweden

## Abstract

Twelve mongrel dogs were randomly allocated into two groups using matched paired-design. Catheters were inserted into the hepatic artery, hepatic vein and the femoral vein, respectively. In the first group, gelfoam supplemented with mitomycin C (MMC) was injected into the hepatic artery, whereas the second group received a hepatic arterial injection of MMC solution alone. Simultaneous blood sampling from the hepatic and femoral veins at regular intervals was performed. MMC concentrations in plasma was determined using high performance liquid chromatography (HPLC) and the pharmacokinetics of MMC were determined. MMC concentrations in hepatic and femoral veins did not differ and no significant difference in pharmacokinetics was found when comparing MMC administration into the hepatic artery with or without gelfoam supplementation. Thus, our results revealed that gelfoam could not delay the clearance of MMC from the liver.


*HPB Surgery*, 1992, Vol. 5, pp. 161–169
Reprints available directly from the publisher
Photocopying permitted by license only

© 1992 Harwood Academic Publishers GmbH
Printed in the United Kingdom

### PHARMACOKINETICS OF MITOMYCIN C FOLLOWING HEPATIC ARTERIAL CHEMOEMBOLIZATION WITH GELFOAM

JIN WEN DING[1], ZAI DE WU[1], ROLAND ANDERSSON[2] and STIG BENGMARK[2]

*1. Department of Surgery, Tongji Hospital, Tongji Medical University, Wuhan 430030, China.*

*2. Department of Surgery, Lund University, S-221 85 Lund, Sweden.*

*(Received 1 June 1991)*

Twelve mongrel dogs were randomly allocated into two groups using matched paired-design. Catheters were inserted into the hepatic artery, hepatic vein and the femoral vein, respectively. In the first group, gelfoam supplemented with mitomycin C (MMC) was injected into the hepatic artery, whereas the second group received a hepatic arterial injection of MMC solution alone. Simultaneous blood sampling from the hepatic and femoral veins at regular intervals was performed. MMC concentrations in plasma was determined using high performance liquid chromatography (HPLC) and the pharmacokinetics of MMC were determined.

MMC concentrations in hepatic and femoral veins did not differ and no significant difference in pharmacokinetics was found when comparing MMC administration into the hepatic artery with or without gelfoam supplementation. Thus, our results revealed that gelfoam could not delay the clearance of MMC from the liver.

KEY WORDS: Mitomycin C, chemoembolization, hepatic artery, liver circulation, pharmacokinetic

### INTRODUCTION

Mitomycin C (MMC) is an antitumor drug with supposed broad spectrum of antitumor activity, first isolated from *Streptomyces caespitosus* by Wakaki *et al.*[1] in 1958. The previously used low-dose regimen caused severe myelosuppression, resulting in only limited use of the drug in the last 20 years. The myelosuppressive adverse effect diminished to a great extent by using a high-dose intermittent schedule[2]. Following advances in understanding of the blood supply of hepatic tumors it was found that liver cancer derives its blood supply almost exclusively from the hepatic artery[3]. This fact focused attention on local treatment of hepatic tumors through the hepatic artery. By selectively increasing the concentrations in tumor tissue, adverse effects on normal cells could be diminished, reducing systemic toxicity and at the same time compensating the low therapeutic index of MMC[4]. This goal could be achieved by bolus intraarterial chemotherapy. MMC, however, proved to present a transient high level in the target tissue, after which it

Address correspondence to: Jin Wen Ding, M.D., M.Sci., Department of Surgery, Lund University, S-221 85 Lund, Sweden was rapidly cleared from the tissue[5,6]. Chemoembolization, including both infarction and prolonged drug action[7], was thought to postpone the tissue clearance. Hepatic arterial chemoembolization (HACE) by gelfoam supplemented with MMC has been used for unresectable liver cancer[8–10]. The advantages of this mode of treatment is that gelfoam embolization can induce ischemia or even necrosis of tumor tissue and creates an anaerobic environment increasing the effect of MMC[11,12]. Moreover, MMC was supposed to be released slowly, thereby reducing the systemic toxicity of MMC. Only limited data on the pharmacokinetics of MMC have, however, been presented due to the lack of sensitive and selective detecting methods. In 1979, Kono *et al.*[13] reported on the use of high performance liquid chromatography (HPLC) for determination of MMC in biological samples.

The purpose of the present study was to compare the pharmacokinetics of MMC after HACE (hepatic arterial chemoembolization using gelfoam and MMC) as compared to hepatic arterial "one-shot" (HAOS), i.e. a bolus injection of MMC alone.

### MATERIALS AND METHODS

Twelve mongrel dogs weighing 12-16 Kg were used for the experiments, randomly allocated into two groups: hepatic arterial "one shot" (HAOS), i.e. a bolus injection of MMC alone, and hepatic arterial chemoembolization (HACE), i.e. MMC and gelfoam together, using matched-paired design. Student's t test was used for statistical analysis. Statistical significance was defined as probability level $p<0.05$.

### *Preparations*

All chemicals used in the experiments were of analytical grade and used without further purification. The pure mitomycin C was purchased from Kyowa Hakkokogyo (Tokyo, Japan).

Gelfoam, 24 $cm^3$ (Wuhan biochemical Co., China), average weight 320 mg, was cut into small pieces of about $1 \times 1$ mm in size using the method described by Bank *et al.*[14], after which the small pieces were sterilized with steam.

MMC, 1 mg/Kg body weight, was dissolved in 20 ml of normal saline and drawn into a glass syringe with gelfoam particles.

### *Experimental Protocol*

Two catheters (I.D. 1.47 mm, O.D. 1.96 mm, dow Corning, Michigan, USA) were inserted into the femoral vein and left or right hepatic artery through the gastroduodenal artery, respectively, under phenobarbital anaesthesia (30 mg/Kg body weight). Methylene blue was injected in the hepatic artery, after which the extent and degree of the blue staining on the surface of the liver was observed. A cardiac catheter (8-F, Shanghai Medical Instrument Co., China) was introduced into the corresponding hepatic vein of the blue stained lobe about 3–4 cm via the jugular vein. An intrahepatic, cord-like mass was then palpable on the surface of the liver, moving when the catheter was partly pulled out, confirming the catheter position in the hepatic vein. Further verification of the position was obtained at autopsy.

MMC or the mixture of gelfoam and MMC was injected into the left or right hepatic artery after catheterization. The syringe was rotated continually during the slow injection (1–2 min.).

### *Blood Sampling*

Blood (1 ml) was simultaneously sampled from the indwelling catheters in the hepatic and femoral veins into heparinized glass tubes prior to injection and at 0, 2, 5, 10, 15, 30, 60, 120, 180, 240, 300 and 360 minutes after injection. The blood samples were centrifuged at 3,000 rpm for 5 minutes, after which red blood cells and plasma were separated and plasma samples frozen at –30 °C until analysis.

### *HPLC Assay*

A modification of den Hartigh's method[15] for plasma MMC extraction and detection was used. 0.5 ml plasma was mixed twice with 5 ml of chloroform/isopropanol (1:1, w/w), shaken for two minutes, and centrifuged at 3,000 rpm for 5 minutes. The organic supernant was transferred into conical glass tubes and evaporated to dryness under nitrogen stream in 40 °C water bath. The residue was dissolved in 100 $\mu$l methanol. A 20 $\mu$l aliquot was used for the liquid chromatography analysis.

The high performance liquid chromatography system consisted of a LC-3 solvent delivery system, SPD-1 ultraviolet detector, CR-1B data processor (all from Shimadzu Co., Japan), YQG-5 $C_{18}$ 4.6×200 mm reverse phase column (Dalian Chromatography Technique Exploiting Co., China). The mobile phase (60% water and 40% methanol) was pumped at 0.8 ml/min. The detector was operated at 365 nm and the retention time was approximately 7 minutes. The standard curve for the drug dissolved in methanol was linear from 5 to 500 ng/ml. The recovery of MMC in plasma was 81 ± 5.2%, and the limit of determination was 50 ng/ml plasma.

### *Pharmacokinetic Data Analysis*

The data on MMC concentrations in plasma was put into an IBM-PC computer operated with a PKBP-N1 pharmacokinetic analysis program package (Dept. of Computer, Army General Hospital of Nanjin Area, China) and depicting the real MMC concentration versus time on a semilogarithmic curve. The curve had a typically biphasic distribution compatible with a two compartment open model. Each set of experimental data fitted to a biexponential equation, $C = Ae^{-\alpha t} + Be^{-\beta t}$, and the pharmacokinetic parameters were calculated from computer-generated coefficients and exponents as described by Wagner[16]. Finally, the theoretical concentration versus the time curve, simulated by the computer, was drawn.

### RESULTS

The theoretical curve simulated by the computer, was very close to the real concentration values. The regression coefficients were 0.9–0.99. No statistically significant differences ($p>0.05$) in MMC pharmacokinetics calculated by MMC concentrations in the hepatic and femoral veins were found neither between the two groups (Table 1, 2) nor within the same group.

**Table 1** MMC pharmacokinetic parameters in HACE group (mean ± SEM)

| *Route* | $T_{1/2\alpha}$ | $T_{1/2\beta}$ | *Vc* | *Vd* | *Cl* | *AUC* |
|---|---|---|---|---|---|---|
| Hepatic vein | 4.41 ± 0.77 | 91.63 ± 5.59 | 0.562 ± 0.056 | 2.277 ± 0.289 | 17.991 ± 2.990 | 65.05 ± 13.22 |
| Femoral vein | 5.70 ± 1.29 | 75.57 ± 7.81 | 0.831 ± 0.127 | 1.944 ± 0.309 | 17.849 ± 2.049 | 61.30 ± 9.57 |

$T_{1/2\alpha}$ (min) Half-time of distribution phase
$T_{1/2\beta}$ (min) Half-time of elimination phase
Vc (L/Kg) Central compartment volume of distribution
Vd (L/kg) Apparent volume distribution
Cl (ml/min/Kg) Total body clearance
AUC (ml/min/kg) Area under the curve

**Table 2** MMC pharmacokinetic parameters in HAOS group (mean ± SEM)

| *Route* | $T_{1/2\alpha}$ | $T_{1/2\beta}$ | *Vc* | *Vd* | *Cl* | *AUC* |
|---|---|---|---|---|---|---|
| Hepatic vein | 4.16 ± 0.58 | 86.02 ± 4.29 | 0.339 ± 0.0p78 | 1.591 ± 0.171 | 13.122 ± 1.528 | 83.19 ± 11.14 |
| Femoral vein | 5.29 ± 0.37 | 54.91 ± 5.81 | 0.680 ± 0.137 | 1.688 ± 0.382 | 21.009 ± 4.417 | 57.53 ± 10.13 |

$T_{1/2\alpha}$ (min) Half-time of distribution phase
$T_{1/2\beta}$ (min) Half-time of elimination phase
Vc (L/Kg) Central compartment volume of distribution
Vd (L/kg) Apparent volume distribution
Cl (ml/min/Kg) Total body clearance
AUC (ml/min/kg) Area under the curve

### DISCUSSION

The pharmacokinetic behavior of MMC administered through a peripheral vein has been studied by several authors[17–21]. Some[18-22] have also investigated the pharmacokinetics of MMC administered in combination with other antineoplastic agents. van Hazel *et al.*[20] reported that other antineoplastic agents did not apparently affect the pharmacokinetic course of MMC, while Verweij *et al.*[19] found that total body clearance of MMC was obviously increased, whereas the area under the curve (AUC) remarkably decreased when MMC was used in combination with other antitumor agents. The reported pharmacokinetics of MMC are somewhat controversial. According to the results of den Hartigh[17], pharmacokinetics of MMC appeared to be non-linear, dose-dependent. van Hazel *et al.*[20] and Schilcher *et al.*[21], however, showed that the pharmacokinetics of MMC administered in different doses up to 60 mg/m$^2$ was not dose-dependent. Hu *et al.*[25] and Gyves *et al.*[26] studied the pharmacokinetics of MMC infused into the hepatic artery, and the local concentration of MMC was found to be 3--4 times higher than that following infusion through a peripheral vein. Systemic toxicity was, however, not reduced by hepatic artery infusion.

MMC has been reported to be eliminated through the kidney, liver, and bile. MMC is eliminated through the kidney by glomerular filtration independent of renal function[20,27,28]. The concentration of MMC in bile was found to be several times higher than in plasma[23], and the elimination of MMC was not influenced even by cholestasis[28,29]. Enzymatic inactivation of MMC was potent especially under anaerobic conditions[30]. Currently, the exact clearance mechanisms of MMC are not clear, but van Hazel *et al.*[29] have stressed the influence of liver function on the elimination of MMC, and the liver has been regarded as a major route of clearance of MMC[22].

In the present study, no statistical difference in elimination could be seen, as calculated by MMC plasma concentrations in the hepatic and femoral veins, even though the half-time tended to be longer in the group with MMC and gelfoam (HACE) as compared to MMC alone (HAOS) infused into the hepatic artery. This indicates that the addition of gelfoam to MMC could not retard the elimination of MMC from the liver. Both in the HACE group and in the HAOS group, the values of the central compartment volume of distribution was about half of the apparent volume of distribution. The distribution of MMC in the circulation and in the systemic tissue was thus equal, and there was no apparent MMC accumulation in tissue. These results are consistent with those of Malviya *et al.*[32]. The difference in the area under the concentration-time curve of MMC in the femoral vein between the two groups was not statistically significant, demonstrating that systemic exposure and toxicity of MMC following both therapies should be the same. Also the concentration-time curves of MMC in the hepatic vein was similar in the two groups, further confirming that the release of MMC in the hepatic vein was the same, supporting the findings of Kato *et al.*[33].

The similar pharmacokinetics of MMC following both therapies might be explained by the following mechanisms. As gelfoam particles only occupied a small part of the 20 ml of MMC solution, a large proportion of the drug could be expected to be in a free state. Also the arterial pressure distal to the embolized arteries reduces, thereby increasing the pressure gradient to the systemic circulation after hepatic arterial embolization, inducing opening of pre-existing collaterals and arterioportal shunts[34–37]. Non-encapsulated MMC might also be metabolized by liver enzymes.

In conclusion, our results show that the pharmacokinetic behavior of MMC was similar both following hepatic arterial chemoembolization using MMC and gelfoam and hepatic arterial injection of MMC alone. This implies that mechanical embolization using gelfoam is not sufficient to prolong MMC clearance from liver tissue.

### *References*

1. Wakaki, S., Marumo, H., Tomika, K. *et al.* (1958) Isolation of new fraction of antitumor mitomycins. *Antibiotics and Chemotherapy*, **8**, 228–240
2. Carter, S.K. (1979) Reflections and prospects. *In* Mitomycin C - Current status and new developments, edited by S.K.Carter and S.T.Crooke, p. 251. New York: Academic Press
3. Breedis, C. and Young, G. (1954) The blood supply of neoplasms in the liver. *American Journal of Pathology*, **30**, 969–985
4. Kato, T. (1983) Selective arterial chemo-embolization with microencapsulated anticancer drugs. *Gan To Kagaku Ryoho*, **10**, 333–339
5. Kato, T., Nemoto, R., Mori, K. and Kumagai, I. (1980) Sustained-release properties of microencapsulated mitomycin C with ethylcellulose infused into the renal artery of the dog. *Cancer*, **46**, 14–21

6. Tsuchiya, S., Mishima, A., Saito, M. *et al.* (1983) Treatment of unresectable hepatocellular carcinoma by arterial infusion of mitomycin C microcapsules (MMC mc). *Nippon Shokakibyo Gakkai Zasshi*, **80**, 185–193
7. Kato, T., Nemoto, R., Mori, H. *et al.* (1981) Arterial chemoembolization with microencapsulated anticancer drug. *Journal of the American Medical Association*, **245**, 1123–1127
8. Yamada, R., Sato, M., Kawabata, M. *et al.* (1983) Hepatic artery embolization in 120 patients with unresectable hepatoma. *Radiology*, **148**, 397–401
9. Yu, Z.L., Jin, J.G., Qu, Q.X. and Wang, B.E. (1986) Chemoembolization for primary hepatic carcinoma. *Journal of Clinical Medicine*, **2**, 79–81 (in Chinese)
10. Nakamura, H., Oi, H., Tanaka, T. *et al.* (1984) Treatment of hepatic tumors by transcatheter chemo-embolization with gelatin sponge. *Gan To Kagaku Ryoho*, **11**, 789–797
11. Kennedy, K.A., Rockwell, S. and Sartorelli, A.C. (1980) Preferential activation of mitomycin C to cytotoxic metabolites by hypoxic tumor cells. *Cancer Research*, **40**, 2356–2360
12. Teicher, B.A., Lazo, J.S. and Sartorelli, A.C. (1981) Classification of antineoplastic agents by their selective toxicities toward oxygenated and hypoxic tumor cells. *Cancer Research*, **41**, 73–81
13. Kono, A., Hara, Y., Eguchi, S. and Tanaka, M. (1979) Determination of mitomycin C in biochemical specimens by high performance liquid chromatography. *Journal of Chromatography*, **164**, 404–406
14. Bank, W.O. and Kerber, C.W. (1979) Gelfoam embolization: a simplified technique. *American Journal of Roentgenology*, **132** 299–301
15. den Hartigh, J., van Oort, W.J., Bocken, M.C.Y.M. and Pinedo, H.M. (1981) High performance liquid chromatography determination of the antitumor agent mitomycin C in human blood plasma. *Analtica Chimica Acta*, **127**, 47–53
16. Wagner, J.G. (1976) Linear pharmacokinetic equations allowing for direct calculation of many needed pharmacokinetic parameters from the coefficients and exponents of polyexponential equations which have been fitted to the data. *Journal of Pharmacokinetics and Biopharmaceutics*, **4**, 443–467
17. den Hartigh, J., Bocken, M.C.Y.M., Gall, H. *et al.* (1982) Pharmacokinetics of mitomycin C (MMC) in man. *Proceedings of the American Association for Cancer Research*, **23**, 126
18. Buice, R.G., Niell, H.B., Sidhu, P. and Gurjey, B.J. (1984) Pharmacokinetics of mitomycin C in non-oat cell carcinoma of the lung. *Cancer Chemotherapy and Pharmacology*, **13**, 1–4
19. Verweij, J., Stuurman, M., Vries, J.D. and Pinedo, H.M. (1986) The difference in pharmacokinetics of mitomycin C, given either as a single agent or as a part of combination chemotherapy. *Journal of Cancer Research and Clinical Oncology*, **112**, 283–284
20. van Hazel, G.A., Scott, M., Rubin, J. *et al.* (1983) Pharmacokinetics of mitomycin C in patients receiving the drug alone or in combination. *Cancer Treatment Reports*, **67**, 805–810
21. Schilcher, R.B., Young, J.D., Ratanatharathorn, V., Karanes, C. and Baker, L.H. (1984) Clinical pharmacokinetics in high-dose mitomycin C. *Cancer Chemotherapy and Pharmacology*, **13**, 186–190
22. van Hazel, G.A. and Kovach, J.S. (1982) Pharmacokinetics of mitomycin C in humans. *Proceedings of the American Association for Cancer Research*, **23**, 125
23. Fujita, H. (1972) Comparative studies on the blood level, tissue distribution, extraction and inactivation of anticancer drugs. *Japanese Journal of Clinical Oncology*, **12**, 151–162
24. Reich, S.D. (1979) Clinical pharmacology of mitomycin C. *In* Mitomycin C - Current status and new developments, edited by Carter, S.K. and Crooke, S.T. p. 243. New York: Academic Press
25. Hu, E. and Howell, S.B. (1983) Pharmacokinetics of intraarterial mitomycin C in humans. *Cancer Research*, **43**, 4474–4477
26. Gyves, J., Ensminger, W. and Stertson, P. *et al.* (1983) Clinical pharmacology of mitomycin C (mito) by hepatic arterial infusion. *Proceedings of American Society of Clinical Oncology*, **2**, 25
27. Schwartz, H.S. and Philips, F.S. (1961) Pharmacology of mitomycin C. II Renal extraction and metabolism by tissue homogenates. *Journal of Pharmacology and Experimental Therapy*, **133**, 335–342
28. Verweij, J., den Hartigh, J., Stuurman, M., Vries, J.D. and Pinedo, H.M. (1987) Relationship between clinical parameters and pharmacokinetics of mitomycin C. *Journal of Cancer Research and Clinical Oncology*, **113**, 91–94
29. den Hartigh, J., McVie, J.G., van Oort, W.J. and Pinedo, H.M. (1983) Pharmacokinetics of mitomycin C in humans. *Cancer Research*, **43**, 5017–5021
30. Schwartz, H.S. (1961) Pharmacology of mitomycin C: III In vitro metabolism by rat liver. *Journal of Pharmacology and Experimental Therapeutics*, **136**, 250–258

31. van Hazel, G.A. and Kovach, J.S. (1982) Pharmacokinetics of mitomycin C in rabbit and human. *Cancer Chemotherapy and Pharmacology*, **8**, 189–192
32. Malviya, V.K., Young, J.D., Boike, G., Gove, N. and Deppe, G. (1986) Pharmacokinetics of mitomycin C in plasma and tumor tissue of cervical cancer patients and in selected tissue of female rats. *Gynecologic Oncology*, **25**, 160–170
33. Kato, T., Nemoto, R., Mori, H., Tanahashi, M. and Tamakawa, Y. (1981) Transcatheter arterial chemoembolization of renal cell carcinoma with microencapsulated mitomycin C. *The Journal of Urology*, **125**, 19–24
34. Eriksson, I. and Sahlstedt, B. (1971) The development of collateral arteries. An angiographic and microscopic study in the rabbit. *Scandinavian Journal of Thoracic Cardiovascular Surgery*, **5**, 265–274
35. Cho, K.J. and Lunderquist, A. (1983) Experimental hepatic artery embolization with gelfoam powder: Microfil perfusion study of the rabbit liver. *Investigative Radiology*, **18**, 189–193
36. Bookstein, J.J., Cho, K.J., Davis, G.B. and Dail, D. (1982) Arterioportal communications: Observations and hypotheses concerning transsinusoidal and transvasal types. *Radiology*, **142**, 581–590
37. Charnsangavej, C., Chuang, V.P., Walace, S., Soo, C.S. and Bower, T. (1982) Angiographic classification of hepatic arterial collaterals. *Radiology*, **144**, 485–494

*(Accepted by L. Blumgart 1 June 1991)*

### INVITED COMMENTARY

It is more than two decades since hepatic artery infusion chemotherapy for tumors of the liver was first used in clinical cases. That therapy was demonstrated to achieve a high drug concentration in the tumor tissue, and it was anticipated that the therapy would enable reduction of systemic adverse reactions. Therefore, the technique was aggressively applied to clinical cases, and good antitumor efficacy has been reported. In the 1970's, transarterial embolization (TAE) with Gelfoam was devised for hepatocellular carcinoma, and a survival rate almost equal to that with surgery was reported, especially in patients with unresectable hepatoma. The effect of TAE was surmised to be derived from its embolization of tumor vessels and subsequent ischemic necrosis of the tumor. However, only a few reports have dealt with the pharmacokinetics of infused anti-tumor agents, including whether or not washout of the drugs is delayed by the decreased blood flow. Dr. Ding studied the pharmacokinetics of MMC after chemoembolization with Gelfoam and concluded that mechanical embolization using Gelfoam is not sufficient to significantly delay MMC clearance from the liver tissue.

However, if we assumed that Gelfoam can retain MMC in it and release of MMC into the blood is prolonged, the pharmacokinetics of MMC in chemoembolization should fit the two-compartment model with a first-order absorption process ($C = -He^{-kat} + Ae^{\alpha t} + Be^{-\beta t}$), because transition of MMC from Gelfoam into the blood flow can be simulated to the absorption process from muscle or the intestine. The apparent release of MMC from Gelfoam suspension is actually very fast; that is, the value of ka is very large, and $-He^{-kat}$ can be considered negligible. Therefore, the pharmacokinetics of MMC can be considered to approximate a biexponential equation. The fact that the curve of the real MMC concentration versus time is close to the theoretical biphasic curve implies that the Gelfoam suspension has no delayed-release effect on MMC. Moreover, the absence of a significant difference in the pharmacokinetics of MMC with or without Gelfoam supplementation indicates that there is no difference in the systemic elimination of MMC, which is chiefly occurred in the liver, between the two groups. However, elongation of the half-life in the embolization group suggests that the ischemia of the liver might be exerting some influence on the elimination of MMC from the liver blood flow and tissue.

Here, "no difference" should be noted. In this experiment, MMC was directly injected into the hepatic artery, so intrinsic clearance (CL = D/AUC) of MMC can be calculated from the data of AUC in femoral vein. From the data shown in this paper, intrinsic clearance of MMC with or without Gelfoam is considered to have no difference. This means no increase of toxicity with supplementation of Gelfoam in the normal liver tissue, and suggests advantageous result for chemoembolization from the aspect of side effects.

It is well known that the arterial blood supply is dominant in hepatic tumors and the pharmacokinetics of antitumor drugs in tumors are surmised to be different from normal hepatocytes, which are also fed by the portal vein. In fact, experiments concluded at our institution by infusing a mixture of doxorubicin and an embolic material into the hepatic artery demonstrated a significant increase in the doxorubicin concentration in tumor tissue compared to the normal liver tissue. Therefore, in order to more accurately investigate the effect of chemoembolization, further studies on the pharmacokinetics of MMC in tumor tissue are needed.

Lipiodol and degradable starch microspheres (DSM) are also used as an embolic material. These substances have a smaller diameter than Gelfoam and many authors have reported the clinical effectiveness of TAE using these materials. Further studies on MMC with these embolic materials and using the method established in this paper are looked forward to.

Yasutsugu Bandai, Yasuo Idezuki
University of Tokyo
Japan

### *References*

1. Ariel, I.M. and Pack, G.T. (1965) Intra-arterial chemotherapy for cancer metastatic to liver. *Arch. Surg.*, **91**, 851–862
2. Provan, J.L., Stokes, J.F. and Edwards, D. (1968) Hepatic artery infusion chemotherapy in hepatoma. *Br. Medical J.*, **3**, 346–349
3. Doyon, D., Mouzon, A., Jourde, A.M. *et al.* (1974) L'embolisation arterelle hepatique dans les tumeurs malignes du foie. *Ann ales de Radiologie*, **17**, 593–603
4. Yamada, R., Nakatsuka, H., Nakamura, K. *et al.* (1979) Transcatheter arterial embolization therapy in unresectable hepatomas - experience in 15 cases. *Acta Hepatol. Jap.*, **20**, 595–603
5. Ekelund, L., Stigsson, L., Jonsson, N. and Sjogren, H.O. (1977) Transcatheter arterial embolization of normal livers and experimental hepatic tumors in the rat. *Acta Radiol. Diagnosis*, **18**, 641–651
6. Bierman, H.R., Byron, R.L.Jr., Kelly, K.H. and Grady, A. (1951) Studies on the blood supply of tumors in man. III. Vascular patterns of the liver by hepatic arteriography *in vivo*. *J. Natl. Cancer Inst.*, **12**, 107–131

7. Konno, T., Maeda, H., Iwai, K. *et al.* (1984) Selective targeting of anti-cancer drug and simultaneous imaging enhancement in solid tumors by arterially administered lipid contrast medium. *Cancer*, **54**, 2367–2374
8. Ensminger, W.D., Gyves, J.W., Stetson, P. and Walker-Andrews, S. (1985) Phase I study of hepatic arterial degradable starch microspheres and Mitomicin. *Cancer Res.*, **45**, 4464–4467

